# A Rare Cause of Apparent Anuria After Caesarean Section: A Case Report

**DOI:** 10.7759/cureus.14400

**Published:** 2021-04-10

**Authors:** Rahul Singh, Abhishek Singh, Rafael Pratts, Ayushi Jain, Madhu Singh

**Affiliations:** 1 Accident and Emergency, Dr. Balwant Singh's Hospital, Georgetown, GUY; 2 Obstetrics and Gynaecology, Dr. Balwant Singh's Hospital, Georgetown, GUY; 3 Urology, Dr. Balwant Singh's Hospital, Georgetown, GUY; 4 Radiology, Mayo Clinic, Jacksonville, USA

**Keywords:** urinary catheter, anuria, caesarian section

## Abstract

Development of anuria in the setting of post-caesarean* *section is a cause of concern for surgeons, and it requires rapid evaluation for detecting its possible etiology and the initiation of appropriate treatment. When the cause of anuria is not immediately apparent in a hemodynamically stable patient, one must rule out other possible differentials as part of the diagnostic process. In this report, we present a rare case of a patient undergoing a trial of labour after a caesarean section, who had a Foley catheter accidentally placed and inflated in the right ureter, instead of the bladder. This led to apparent anuria, which was not identified until the patient's caesarean section had been completed. We also seek to shed light on a method for safely removing a ureteral Foley with a blocked inflation channel.

## Introduction

Bladder catheterisation is frequently employed in obstetrics and gynaecology as a measure to prevent bladder injuries during procedures such as caesarean sections. We report a case of accidental placement of the Foley catheter in the ureter while attempting to place it in the bladder. 

Although some cases of accidental ureteric catheterisation have been reported, most of them have been documented in patients with a neurogenic bladder [1,2]. As late as 2017, such case reports were rare and only 20 such cases had been documented [1]. Among these, a case involving inflation of the Foley balloon in a ureter is even more uncommon [3], with a few other instances having been reported as occurring in the peripartum period [4] in an otherwise normal ureter, thereby making this case an unusual incidence of apparent anuria.

Apparent anuria in a patient immediately following a caesarean section is not a trivial matter, because apart from simpler causes like a blocked catheter or a defective catheter, one also has to consider more serious causes such as ureteral injury, ligation or transection, renal causes, and pre-renal causes such as hypovolemia in cases with inordinately heavy blood loss.

## Case presentation

A 22-year-old woman underwent a repeat caesarean section on request as she did not wish to proceed with the trial of labour after her previous caesarean section. A Foley catheter was inserted while the patient was in labour as is routine for all patients in labour with a history of a previous caesarean section. The midwife responsible for the insertion of the Foley mentioned urine drainage at insertion but failed to record further output. The patient was transferred to the operating theatre for the caesarean section without anyone noticing the absence of urine draining into the urine bag.

Once the surgery was completed and the abdomen was closed, the empty urine bag was noticed and brought to the attention of the operating team. Attempts were made initially to flush the Foley through the inflation channel, without any success. Efforts were then made to deflate the Foley bulb, using routine methods of Foley removal, such as aspiration through the inflation channel to deflate the bulb; however, these measures also proved unsuccessful. The catheter was then cut to disable the valve in the inflation channel to facilitate drainage, about 6 cm from the meatus, but the bulb still could not be drained.

Once the catheter was cut and the inflation channel was visible, an attempt was made to insert a needle attached to a syringe to attempt aspiration of the fluid through the inflation channel, which also proved unsuccessful. At this stage, we had to step back and reevaluate the problem. We decided to perform a vaginal examination to try to palpate the Foley so that we could estimate where it was inflated and thereby use an appropriate method to solve the problem. On vaginal examination, the Foley bulb was found palpated in the upper right fornix of the vagina, which was highly indicative of it being in the right ureter.

Investigations

A rigid 6/7.5 Fr ureteroscope (Richard Wolf GmbH, Knittlingen, Germany) was inserted into the bladder along the Foley catheter, and the Foley bulb was seen in the distal part of the right ureter, just beyond the meatus where it had been inflated. 

Differential diagnosis

When encountering a patient who has just undergone a caesarean section and is found to be “anuric”, one first considers likely causes such as a blocked catheter, a defective catheter, or a catheter that has been improperly placed in the urethra and does not quite reach the bladder. One also has to consider more serious causes such as ureteric injury, ligation or transection, renal impairment, and hypovolemia. In our case, what we found was a rare cause, which had occurred for the first time in our 25-year experience in the medical field.

Treatment

Under ureteroscopic guidance, attempts to puncture the balloon were then made using a three-pronged stone removing forceps, and a 145 cm 0.035” extra-stiff guidewire, which proved unsuccessful. At this stage, we decided to try a rigid 1.2 mm lithotripsy probe, which was inserted through the ureteroscope; the Foley bulb was punctured with the probe and the Foley catheter was removed.

Once the Foley catheter was removed, the ureteroscope was inserted once again and the entire length of the ureter was checked for injuries. A small mucosal abrasion was identified at the site of balloon inflation. Under fluoroscopic guidance, a guidewire and a 6 Fr double J stent measuring 26 cm were placed in the ureter. The guidewire and ureteroscope were then withdrawn and removed. A Foley catheter was placed in the bladder and the patient was placed on continuous drainage, leaving the ureteral stent in situ.

**Figure 1:**
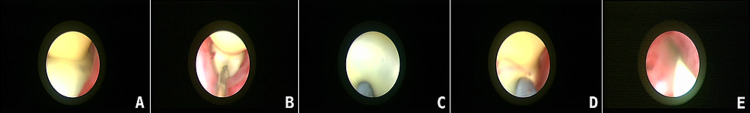
Images obtained during the cystoscopy performed using a ureteroscope depicting the bulb of the Foley catheter in the right ureteral ostium, unsuccessful attempts to puncture the Foley bulb, and our final successful attempt A. The Foley catheter inflated in the right ureteral ostium, with the Foley catheter in the upper field B. Three-pronged lithotripsy forceps indenting the bulb of the Foley as part of the initial attempt to puncture it C. The guidewire indenting the Foley bulb in the lower field D. The rigid lithotripsy probe indenting the bulb; the probe eventually punctured the bulb E. The superficial abrasion after the Foley catheter was removed, with the guidewire also seen, which was used for stent placement

A ureteroscopy was also performed at the end of the procedure to visually inspect the entire length of the ureter for any further damage (Video 1, Figure 1).

**Video 1:** The video shows the procedure of puncturing the bulb of the Foley catheter by using a lithotripsy probe

Outcome and follow-up

The patient was discharged 48 hours after the caesarean section with a Foley catheter on drainage and ureteral stent in situ. She returned for a post-caesarean section follow-up after seven days. The Foley catheter was removed 14 days after the surgery. The ureteric stent was removed four weeks after the surgery. After the removal of the stent, ultrasound examinations were performed at the follow-ups at one week, four months, and nine months, all of which were found to be normal. Of note, there were no renal changes, such as hydronephrosis, to indicate ureteric stricture formation or vesicoureteral reflux. We also did not find any congenital predisposing factors, such as ureteral duplication and ectopic ureters.

## Discussion

Every patient in our practice undergoing a caesarean section has a Foley catheter placed in the bladder to prevent bladder injury during the surgery, and this is usually removed a few hours after the surgery when the patient is ambulatory and can void on her own. While placing the Foley catheter in this particular case, our midwife failed to assess the length of the catheter remaining outside the urethral meatus before inflating it. If she had done so, she would have likely realized that the catheter was inflated above the bladder, rather than within it [2]. This was the first case in our hospital of inadvertent ureteric catheterisation by a Foley catheter that was meant to drain the bladder. We believe an accidental event such as this should form part of the differential diagnosis of apparent anuria in a patient after Foley catheterisation of the bladder as it will in our practice.

Accidental ureteric catheterisation is a rare complication, more commonly seen in certain patient populations, particularly those with contracted bladders, such as patients with neurogenic bladders, and those with long-term catheterisation [1,5]. In our case, physiologic changes in pregnancy, such as dilation of the ureteric orifices, may have predisposed the patient to inadvertent ureteric catheterisation [2,6]. However, this increased risk in obstetric patients has been refuted by some researchers who have found that the lower one-third of the ureter does not undergo dilation in pregnancy [7]. Other populations that may be prone to the accidental ureteric placement of a Foley catheter meant for bladder drainage are those with congenital anatomic abnormalities such as a double ureter, ectopic ureters, or those with iatrogenic factors such as a change in anatomy after a retropubic colposuspension [8,9].

In our case, when we realized that the Foley catheter was not draining any urine, attempts were made to remove the non-deflating Foley catheter using methods previously described related to such a scenario, such as cutting the catheter to disable the valve in the inflation channel [10]. When the catheter is cut, precautions must be taken to secure the catheter with an instrument, such as an artery forceps, to ensure that it does not slip into the bladder [11]. It has also been mentioned in the literature that a guidewire can be inserted through the inflation channel and the balloon can be punctured and drained. However, it has proved difficult for other practitioners and may not have been possible in our case where the inflation channel appeared not to be patent [12-14] as was indicated by our failure to aspirate through the inflation channel during the previous maneuver. This apparent blockage may have arisen because, as reported by other authors faced with a similar situation [4], the Foley bulb when inflated in the ureter is asymmetrical in relation to the main catheter and its lumen. This conjecture was tested by the aforementioned authors ex vivo [4] and was confirmed by ourselves by using an endotracheal tube as a ureteric model and inflating a Foley catheter balloon inside it. This asymmetrical relationship may cause a kink in the tip distal to the bulb, thereby preventing aspiration, drainage, and passing of a guidewire through the inflation channel to rupture the balloon [4]. 

Based on our review of the literature on ureteric injuries secondary to a Foley catheter being placed and inflated in the ureter [5], a CT scan or an intravenous urography examination could have been done to exclude a ureteric rupture. However, since the entire length of the ureter from the meatus to the renal pelvis had been visualized using ureteroscopy performed by our urologist on the case and as he was certain that there was no breach, he did not consider it necessary to perform the imaging with contrast. It has also been mentioned in the literature that patients with inflation of the balloon in the ureter may develop strictures and should be followed up, either by ultrasound or CT intravenous pyelogram to image the upper tract [1]. In our case, the follow-up was done by ultrasound at one week, four months, and nine months after the stent removal.

Preventative guidance measures proposed by authors who have encountered similar complications include assessing the length of the Foley remaining outside the external meatus after inflation of the balloon [2]. Inserting more than the required length of the Foley catheter into the bladder should be avoided, especially in patients who may have hydroureteronephrotic changes. Relevant to this case, such changes are supposedly common in pregnant and immediate postpartum patients [2,6].

## Conclusions

Accidental ureteric Foley catheterisation could form part of the differential diagnosis in an apparently anuric, catheterised patient. Ureteric catheterisation could be avoided by ensuring that the length of the catheter inside the bladder does not exceed 6-8 cm before inflation; one could use the length of the Foley catheter that remains outside the urethral meatus as a guide for this. In cases of accidental ureteric catheterisation, one could use a rigid lithotripsy probe to puncture the balloon of the retained Foley catheter. When cutting an inserted Foley catheter, the cut end of the catheter must be secured with an instrument, to ensure it does not prolapse into the bladder, which may necessitate cystoscopic removal.
